# Low cytotoxicity and clastogenicity of some polymeric aminophosphonate derivatives

**DOI:** 10.1080/13102818.2014.901676

**Published:** 2014-06-04

**Authors:** Anton Kril, Margarita Topashka-Ancheva, Any Georgieva, Ivan Iliev, Tsvetelina Gerasimova, Ivanka Kraicheva, Ivelina Tsacheva, Anita Bogomilova, Elitsa Vodenicharova, Kolio Troev

**Affiliations:** ^a^ Bulgarian Academy of Sciences, Institute of Experimental Morphology, Pathology and Anthropology with Museum, Sofia, Bulgaria; ^b^ Bulgarian Academy of Sciences, Institute of Biodiversity and Ecosystems Research, Sofia, Bulgaria; ^c^ Bulgarian Academy of Sciences, Institute of Polymers, Sofia, Bulgaria

**Keywords:** poly(aminophosphonate)s, human epithelial cancer, cell lines, safety testing

## Abstract

Poly(oxyethylene aminophosphonate)s synthesized on the basis of biodegradable poly(phosphorester)s and Schiff bases were tested *in vitro* for antitumor activity against a panel of six human epithelial cancer cell lines, for cytotoxicity to mouse fibroblast cells and *in vivo* for clastogenicity and antiproliferative effects. The polymers showed lower cytotoxicity, both *in vivo* and *in vitro* and lower clastogenicity *in vivo* than the corresponding low-molecular aminophosphonates. The biological activities of the tested polymers correlate with their low *in vitro* antitumor activity.

## Introduction

Aminophosphonic acid derivatives have attracted intense interest owing to their important application in medicine and agrochemistry.[1,2] α-aminophosphonates are considered to be phosphorus analogues of natural amino acids.[3] Because of the structural similarity to the α-aminocarboxylic acids, α-aminophosphonates act as excellent mimetics of natural substrates and are the most active inhibitors of specific enzymes and regulators of metabolic processes.[4] Bisphosphonates inhibit bone resorption by selective adsorption to mineral hydroxyapatite surfaces and are extremely useful in the treatment of cancer-induced bone disorders.[4,5] Aminophosphonates and their polymeric analogues are used as radiopharmaceuticals for diagnostic imaging and therapeutic delivery of radiation to a specific tissue.[4] Aminophosphonate complexes with metal ions serve as contrast agents or paramagnetic shift reagents for NMR active cations in medical diagnostic procedures.[4] Many compounds of this class have found application as antibacterial, antiviral and antitumor therapeutics.[3,6]

One of the promising strategies to improve the therapeutic efficiency of the drugs involves their conjugation with biodegradable and biocompatible polymers.[7] The polymer–drug conjugates have enormous potential to avoid major disadvantages of the conventional drug therapy, such as the toxic side effects, the low solubility in water and the short biological half-life of the agents.[8,9] The incorporation of biologically active aminophosphonate molecules to biodegradable polymer carriers, like poly(phosphorester)s, offers possibility for the preparation of polymer–drug conjugates with phosphorus ester linkages in the backbone that can degrade into biologically compatible components under physiological conditions. Earlier, on the basis of polymeric H-phosphonates and Schiff bases, we synthesized poly(aminophoshonate)s (**1–4**), which consist only of aminophosphonate units with potential biological activity and non-toxic poly(ethylene glycol) links.[10,11] Their synthesis, spectroscopic characteristics and *in vitro* antitumor activity against a panel of four human tumour cell lines (CLs), namely HL-60, its multi-drug-resistant sub-line HL-60/Dox, LAMA-84 and K-562, were described.[10,11] In this work we report on the *in vitro* antitumor activity of the above-mentioned poly(oxyethylene aminophosphonate)s against several human epithelial cancer CLs, as well as on their *in vitro* and *in vivo* safety testing. The low-molecular analogues of these poly(aminophosphonate)s have been already studied for cytotoxicity against the same tumour CLs and their safety has been evaluated both *in vitro* and *in vivo*.[12,13]

## Materials and methods

Poly(oxyehtylene aminophosphonate)s **1–4** were obtained according to previously described procedures.[10,11] The Schiff bases, *N*,*N*-dimethyl-*N*′-furfurylidene-1,3-diaminopropane and *N*-(4-dimethylaminobenzylidene)-*p*-toluidine were prepared following well-known procedures.[14,15] The poly(oxyethylene H-phosphonate)s were synthesized *via* a polytransesterification reaction of dimethyl H-phosphonate and poly(ethylene glycol)s with average molecular weight 200 Da (PEG 200) and 600 Da (PEG 600).[16,17] Dimethyl H-phosphonate, poly(ethylene glycol)s, furfural, 4-dimethylaminobenzaldehyde, *N*,*N*-dimethyl-1,3-diaminopropane and *p*-toluidine were purchased from Fluka Chemie GmbH, Buchs, Switzerland.

### *In vitro* investigations

#### Antitumor activity

The antitumor activity testing was performed on cell cultures from several CLs derived from human epithelial tumours using the MTT test.[18] CLs from ductal carcinoma of the breast (MCF-7 and MDA-MB-231- with low and high metastatic potential, respectively), HBL-100 (colostrum derived myoepithelial cells, expressing polyoma virus large T-antigen), hepatocellular carcinoma (HepG2), colon carcinoma (HT-29) and the CL HeLa- cervical carcinoma, were used in all experiments. The CLs used were routinely grown as monolayers in 75 cm^2^ tissue culture flasks (Corning) in high-glucose (4.5‰) Dulbecco's Modified Eagle's Medium (DMEM) (Sigma), supplemented with 10% fetal calf serum (Gibco) and antibiotics in usual concentrations. Cultures were maintained at 37.5 °C in a humidified atmosphere and 5% CO_2_. The trypsinized tumour cells were adjusted to a density 1 × 10^5^ cells/ml culture medium and plated (100 μl/well) in 96-well flat-bottomed microplates (Orange Scientific). The cells were allowed to adhere for 24 hours before treatment with test compounds dissolved in dimethyl sulfoxide (DMSO), further diluted in culture medium to reach the desired test concentrations. A concentration range from 1 to 0.0681 mg/ml (six wells per concentration) was applied for 24 hours. The DMSO concentration never exceeded 1% (v/v). The referent antineoplastic drug Doxorubicin hydrochloride (Lemery) was used as a positive control substance. The MTT (3-(4,5-dimethylthiazol-2-yl)-2,5-diphenyltetrazolium bromide) solution (5 mg/ml in PBS) was added (100 μl/well), and plates were incubated for 3 hours at 37.5 °C in a humidified atmosphere and 5% CO_2_. The MTT-formazan crystals were dissolved by adding 100 μl/well of an absolute ethanol/DMSO (1:1 v/v) solution and the absorption was registered using a microplate reader (TECAN, Sunrise TM, Groedig/Salzburg, Austria) at 580 nm. All experiments were performed in triplicate. Cytotoxic activities were expressed as IC_50_ values (concentrations required for 50% inhibition of cell growth), calculated using non-linear regression analysis (GraphPad Prizm5 Software).

#### Safety testing in vitro

The *in vitro* safety testing was performed as described by Borenfreund and Puerner [19] and the latest modification of the validated Balb/c 3T3 (clone 31) Neutral Red Uptake Assay (3T3 NRU test).[20] Balb/c 3T3 mouse embryo cells were grown as monolayers in 75 cm^2^ tissue culture flasks in low-glucose (1‰) DMEM (Sigma), supplemented with 5% fetal calf serum and antibiotics. Cultures were maintained at 37.5 °C in a humidified atmosphere and 5% CO_2_. Cells were plated at a density of 1 × 10^4^ cells in 100 μl culture medium in each well of 96-well flat-bottomed microplates and allowed to adhere for 24 hours before treatment with test compounds, dissolved in DMSO and further diluted in culture medium. A wide concentration range was applied (from 1 to 0.0681 mg/ml; dilution factor of ^6^√10 = 1.47). After 24 hour treatment Neutral Red containing medium was applied for three hours, the cells were washed and ethanol/acetic acid desorbing solution was added. The optical density was measured by a microplate reader at 540 nm. The statistical analysis included application of One-way ANOVA followed by Bonferroni's *post hoc* test and *p* < 0.05 was accepted as the lowest level of statistical significance.

### *In vivo* studies

Male and female laboratory mice ICR (2n = 40) (♂♀ = 53) weighting 20 ± 1.5 g were supplied from the Slivnitza animal breeding house of the Bulgarian Academy of Sciences, Sofia. Animals were kept at standard conditions at temperature 20 °C–22 °C, photoperiod 7 am to 7 pm, free access to standard animal food for laboratory animals – “Rodents” (produced by Vitaprot-Ltd., Kostinbrod, Bulgaria, according prescription 456-1-12) and water.

The experiments were conducted according to approved protocols, and in compliance with the requirements of the European Convention for Protection of Vertebrate Animals used for experimental and other Specific Purposes and the current Bulgarian laws and regulations.

Poly(aminophoshonate)s **2–4** (10 and 100 mg/kg body weight) dissolved in DMSO were injected intraperitoneally (i.p.) only once. Control groups of mice were treated i.p. with 0.9% NaCl and DMSO (0.01 mL/g b.w.).The cytogenetical analysis was performed according to the protocol described by Preston et al. [21]. Mitomycin C (3.5 mg/kg) (Sigma EC No 200-008-6) was selected as a positive control substance – a clastogenic agent with proven genotoxic effect, damaging DNA matrix *via* alkylation. A sufficient number of metaphase chromosomes suitable for cytogenetic analysis were achieved after injection of the mitotic inhibitor colchicine (Sigma) (0.04 mg/g b.w.) one hour before bone marrow cell isolation. Animals were euthanized by diethyl ether, bone marrow cells were flushed from femur and hypotonized in a 0.075 M potassium chloride for 15 min. at 37 ºC. Thereafter, the cells were fixed in cold methanol:glacial acetic acid (3:1), resuspended, dropped on precleaned cold wet slides, air dried and stained in 5% Giemsa solution (Sigma Diagnostic). Up to 50 well-scattered metaphase plates were analysed from each animal by light microscopy (Cetopan Reichert, Austria) at magnification × 1000.

The main types of aberrations–breaks, fragments, exchanges (centromere/centromeric fusions, telomere/telomeric fusions) and pericentric inversions were separately scored. The frequencies of chromosomal aberrations were determined for each animal. The mean ± SEM for each group was calculated and the data were statistically evaluated by analysis of variance using Student's *t-*test.

## Results and discussion

**Figure 1.**
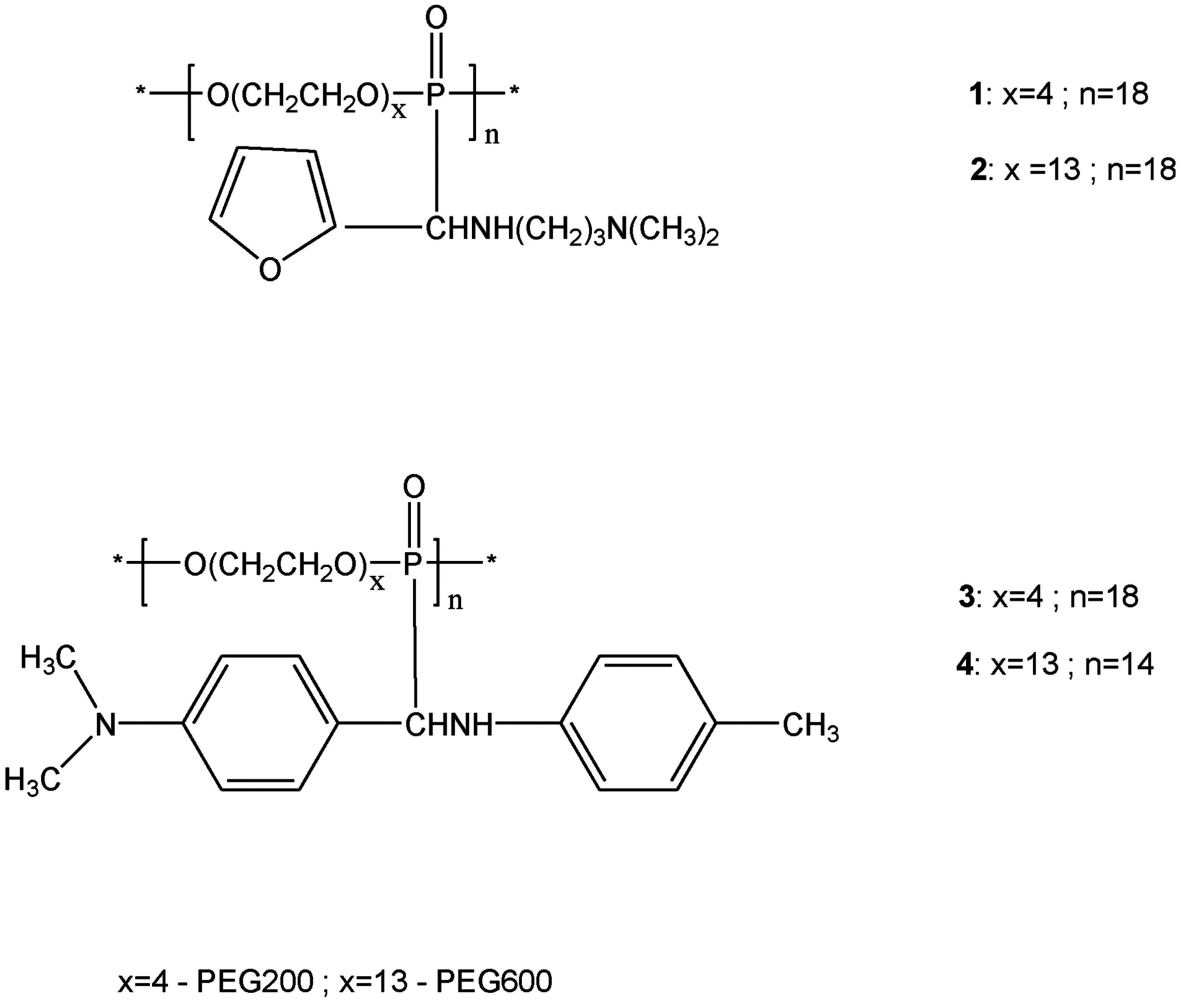
Poly(oxyethylene aminophosphonate)s **1–4**: repeating units.

Poly(oxyehtylene aminophosphonate)s **1–4** (Figure 1) were synthesized through an addition of poly(oxyehtylene H-phosphonate)s to the Schiff bases, *N*,*N*-dimethyl-*N*′’-furfurylidene-1,3-diaminopropane [11] and *N*-(4-dimethylaminobenzylidene)-*p*-toluidine,[10] respectively.

### *In vitro* antitumor activity

The poly(oxyethylene aminophosphonate)s **1–4** were tested for antitumor activity against a panel of six cancer CLs, representative of some important types of human tumours. The tested polymers induced concentration-dependent antiproliferative effects after 24 hour exposure which enabled the construction of concentration response curves (not shown) and the calculation of the corresponding IC_50_ values summarized in Table 1.

**Table 1.** Comparative cytotoxic activity of polymers **1–4** versus referent substance Doxorubicin in a panel of human tumour cell lines after a 24-hour treatment (MTT-dye reduction assay).

	Mean IC_50_ values ± SD (mg/ml)^a^				
Cell lines	1	2	3	4	Doxorubicin
HBL-100	0.831 ± 0.0395	>1	0.894 ± 0.0789	>1	0.14 ± 0.011
MCF-7	0.601 ± 0.0716	>1	0.610 ± 0.1050	>1	<0.068
MDA-MB-231	>1	0.728 ± 0.0991	0.822 ± 0.0552	0.670 ± 0.0379	<0.068
HeLa	0.332 ± 0.0472	>1	0.918 ± 0.0214	>1	<0.068
HepG2	0.916 ± 0.0419	0.572 ± 0.0244	0.635 ± 0.0040	0.665 ± 0.0520	<0.068
HT-29	>1	0.608 ± 0.0057	0.786 ± 0.0965	0.911 ± 0.0070	0.58 ± 0.013

^a^Values are means ± standard deviation from three consecutive experiments.

All polymers were less active in comparison with the referent anticancer drug Doxorubicin. The most prominent antiproliferative effect was observed after treatment of cervical carcinoma CL HeLa with the poly(aminophoshonate) **1**. The poly(aminophoshonate)s **1** and **3** appeared to be more active than the corresponding analogues with longer PEG moiety (**2** and **4**) against the CL HBL-100, MCF-7 and HeLa. Our previous study indicated that the low-molecular weight analogue of polymers **1** and **2,** *N*,*N*-dimethyl-(*N*′-methyl(diethoxyphosphonyl)-(2-furyl))-1,3-diaminopropane, exerts very high antitumor activity *in vitro* to the CL derived from human hepatocellular carcinoma (HepG2).[12] The observed effect was comparable to those of the standard cytostatic drug doxorubicin used as a positive control. The investigated polymeric forms of this compound showed very low antiproliferative activity to this CL. Similarly, the aminophosphonate *p*-(*N*-methyl(diethoxyphosphonyl)-(4-dimethylaminophenyl))toluidine showed higher activity against the CL Hep G2, MCF-7 and HeLa, as compared to the corresponding polymeric forms **3** and **4**. It could be proposed that the higher molecular weight of these polymeric substances is a prerequisite for the hampered penetration through the cell membrane and limited access to the intracellular target sites. More importantly, the interactions of PEGs with biological macromolecules present in fetal calf serum, added to the cell culture medium should also be taken into consideration. In fact, studies from late nineties of the previous century have shown formation of PEG precipitates with immunoglobulins, α_1_-antitrypsin and human serum albumin.[22] Recent studies [23] revealed molecular mechanisms of PEG binding to bovine serum albumin (BSA). It has become apparent that low-molecular weight PEGs induce significant unfolding of BSA molecule, leading to exposure of tryptophan residues, followed by strong physical adsorption of PEG on the hydrophobic core of the protein along with surface adsorption to BSA. Thus, the processes described lead to a diminution of free active molecules in the culture medium and, hence, to a lower *in vitro* antitumor activity.

### *In vitro* safety testing

**Figure 2.**
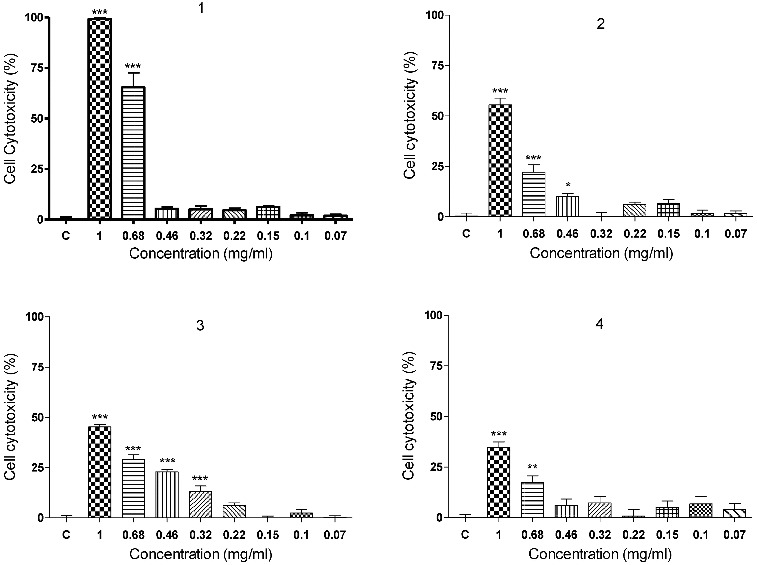
*In vitro* cytotoxicity of compounds **1–4** on cultures from cell line Balb/c 3T3, clone 31 (3T3 NRU test). C: vehicle treated cell cultures (negative control); *p < 0.05; **p < 0.01; ***p < 0.001, compared to negative control.

The results from the validated Balb/c 3T3 (clone 31) Neutral Red Uptake Assay (3T3 NRU test) revealed a dose-dependent cytotoxic activity of polymers **1–4** (Figure 2). The poly(oxyethylene aminophosphonate)s **1** and **2**, obtained from the Schiff base *N*,*N*-dimethyl-*N*′-furfurylidene-1,3-diaminopropane exerted higher cytotoxicity than the polymers **3** and **4**, prepared on the basis of the Schiff base *N*-(4-dimethylaminobenzylidene)-*p*-toluidine. The polymer **1** (IC_50_ = 0.628 ± 0.039) was significantly more toxic (*p* < 0.001) than its analogue **2** with a longer PEG moiety (IC_50_ = 0.935 ± 0.058).

### Clastogenic and antiproliferative effects *in vivo*

The results from the cytogenetical analysis are presented on Table 2. These results point out that the polymer **2**, applied at a concentration of 10 mg/kg showed low clastogenic effect. Only 2.89% ± 0.73% metaphases with aberrant chromosomes were observed 24 hours after treatment. In samples obtained 48 hours after treatment the percentage of aberrant mitoses was 3.81 ± 0.45, but statistically significant difference between the two experimental groups was not evident (*p* > 0.05). The dose of 100 mg/kg did not provoke an increase of the number of bone marrow cells with chromosomal rearrangements, compared to the lower (10 mg/kg) dose. The reported differences in the absolute values of the percentage of aberrant metaphases in samples 24 and 48 hours after treatment were not statistically significant. In general, centromere/centromeric fusions prevailed, while the numbers of breaks and the fragments were approximately equal. The obtained data about the clastogenic effect of polymer **2** showed much lower values than those obtained after treatment of ICR mice with the alkylating agent Mitomycin C (Table 2).

**Table 2.** Clastogenic effect and proliferative activity of bone marrow cells of ICR line laboratory mice after i.p. treatment with poly(oxyethylene aminophosphonate)s **2–4**.

			Type of chromosome aberrations														
					Rearrangements				Statistical significance					Statistical significance			
Compounds and doses	Time after treatment	Number of metaphases scored	Breaks	Fragments	c/c	t/t	c/t	Percentage of cells with aberrations (X ± SEM)	a	b	c	d	Mitotic index (‰) (X ± SEM)	a	b	c	d
**(2)**	24 h	395	3	3	4	0	0	2.89 ± 0.73	***				9.05 ± 1.28	***	***	***	
10 mg/kg	48 h	339	0	3	10	0	0	3.81 ± 0.45	***	***			12.40 ± 1.45	***	***	***	
**(2)**	24 h	381	7	2	4	0	0	3.39 ± 0.35	***	***			3.79 ± 0.62	***	***		
100 mg/kg	48 h	400	0	6	15	0	0	5.25 ± 0.75	***	***			2.38 ± 0.54	***	***		
**(3)**	24 h	400	4	1	4	0	0	2.25 ± 0.25	***		***		8.51 ± 0.63	***	***		*
10 mg/kg	48 h	400	5	2	5	0	0	3.00 ± 0.53	***	**	**		11.54 ± 1.38	***	***		
**(3)**	24 h	400	5	5	10	0	0	4.75 ± 0.36	***	***			6.84 ± 0.32		***		***
100 mg/kg	48 h	400	4	6	10	0	1	5.50 ± 0.5	***	***			9.22 ± 0.43	***	***		
**(4)**	24 h	400	3	3	4	0	0	2.25 ± 0.25	***		***		12.99 ± 0.78	***	***	***	
10 mg/kg	48 h	400	3	3	4	0	0	2.75 ± 0.44	***		***		11.42 ± 0.45	***	***		
**(4)**	24 h	400	1	10	10	2	0	5.75 ± 0.96	***	***			8.44 ± 1.11	**	***		
100 mg/kg	48 h	400	8	3	17	1	1	5.25 ± 0.37	***	***			10.28 ± 0.68		***		
**Mit. C**	24 h	200	17	30	7	1	0	30.5 ± 2.36		***			5.49 ± 0.19		***		
3.5 mg/kg	48 h	400	17	24	20	0	0	15.8 ± 0.81		***			7.29 ± 0.34		***		
**Control**	24 h	700	4	0	4	0	0	1.14 ± 0.34	***				20.06 ± 1.38	***			**
0.9% NaCl	48 h	500	0	0	3	0	0	0.6 ± 0.3	***				16.88 ± 0.56	***			
DMSO	24 h	500	1	1	4	0	0	1.40 ± 0.30	***				15.14 ± 0.46	***	**		
	48 h	500	2	0	2	0	0	0.80 ± 0.32	***				12.47 ± 1.07	***	**		

Note: c/c – centromere/centromeric fusion; t/t – telomere/telomeric fusion; c/t – centromere/telomeric fusion.

Statistics: Student's *t*-test; *p < 0.05; **p < 0.01; ***p < 0.001.

a – compared to Mitomycin C; b – compared to control; c – compared to dose 100 mg/kg; d – 24 h compared to 48 h.

The data about the antimitotic activity showed that the studied polymer **2** strongly suppressed proliferative processes in bone marrow cells *in vivo*. The mitotic index of bone marrow cell populations in the treated mice was only 3.79‰ at the 24th hour and 2.38‰ at the 48th hour after i.p. injection of the animals with **2** (100 mg/kg). Surprisingly, the antimitotic action of **2** in this experimental group was stronger than the effect of Mitomycin C.

The obtained results correlate with the data about cytotoxic activities of the studied substances in Balb/c 3T3 cell cultures.

The polymer **3** applied at a dose of 10 mg/kg showed negligible adverse effect on the structure of chromosomes. Only 2.25% at 24 hours and 3.00% at 48 hours after injection of the analysed metaphases contained chromosomes with damaged structure. In samples from animals treated with 100 mg/kg a significant increase of cells with damaged chromosomes has been found in comparison with samples from mice, treated with lower concentration. The absolute values were 4.75% ± 0.36% (*p* < 0.001) and 5.50 ± 0.50 (*p* < 0.01) for samples obtained 24 and 48 hours after treatment, respectively.

In the two concentrations applied the polymer **3** showed significantly lower chromosome damaging effect, compared to Mitomycin C. The clastogenicity values observed in samples from the experimental group treated with 10 mg/kg were statistically insignificant (*p* > 0.05) as a quantity and type of chromosomal aberrations, compared to untreated control animals.

These results suggest that lower doses (10 mg/kg) of the investigated polymer are not genotoxic (Table 2).

The antimitotic activity of **3** was also calculated. As a result of the action of **3**, applied in concentrations of 10 mg/kg and 100 mg/kg, significantly lower mitotic activity, compared to normal bone marrow cells was counted. The lowest values were reported in the experimental group treated with a dose of 100 mg/kg, 24 hours after injection of the polymer. These results are consistent with the obtained results from the *in vitro* cytotoxicity study.

The clastogenicity of **4** at a concentration of 10 mg/kg was very low. The values of aberrant mitoses did not differ significantly from those observed in normal bone marrow cells (*p* > 0.05). The highest percentage of metaphases with aberrations was 2.75 ± 0.44%, 48 hours after inoculation of the polymer.

The analysis of the experimental data, however, indicated that this polymer compound applied at a concentration of 100 mg/kg induced well-defined dose-dependent clastogenic effect. In the treated animals, twice as higher percentage of bone marrow cells with aberrant chromosomes were recorded, in comparison with the group treated with 10 mg/kg (*p* < 0.001). In these specimens centromere/centromeric fusions prevailed – from 52% at the 24th hour to 62% at the 48th hour after application.

The antimitotic activity of **4** was moderate. The lower dose – 10 mg/kg inhibited the intensity of cell division, expressed as mitotic index values – 12.00±0.78‰ at the 24th hour and 11.42‰ at the 48th hour. The corresponding values for untreated controls were 20.06 ± 1.8‰ and 16.88 ± 0.56‰, 24 and 48 hours, respectively.

## Conclusions

It could be concluded that the three studied *in vivo* polymers possess low clastogenic activity. In the applied concentrations they inhibit cell division, but do not completely impair the proliferative pool of normal bone marrow cell populations. These properties and the established antitumor activity on certain malignant CLs *in vitro* support the need to extend the studies on the biological activity of these polymers to other living model systems.
